# Shifts in the vaginal microbiota of nulliparous ewe lambs following initial reproductive handling and estrus synchronization

**DOI:** 10.1093/jas/skag184

**Published:** 2026-06-05

**Authors:** Edgar L Reinoso-Peláez, Ignacia Beltrán De Heredia, María Saura, Carmen González, Manuel Ramón, Magdalena Serrano

**Affiliations:** Departamento de Mejora Genética Animal, Instituto Nacional de Investigación y Tecnología Agraria y Alimentaria (INIA-CSIC), Madrid, 28040, Spain; Instituto Vasco de Investigación y Desarrollo Agrario, Alianza Vasca para la Investigación y la Tecnología (Neiker-BRTA), Vitoria-Gasteiz, 01080, Spain; Departamento de Mejora Genética Animal, Instituto Nacional de Investigación y Tecnología Agraria y Alimentaria (INIA-CSIC), Madrid, 28040, Spain; Laboratorio de Biotecnología Acuática, Instituto de Investigaciones Marinas (IIM-CSIC), Vigo, 36208, Spain; Departamento de Mejora Genética Animal, Instituto Nacional de Investigación y Tecnología Agraria y Alimentaria (INIA-CSIC), Madrid, 28040, Spain; Departamento de Mejora Genética Animal, Instituto Nacional de Investigación y Tecnología Agraria y Alimentaria (INIA-CSIC), Madrid, 28040, Spain; Departamento de Mejora Genética Animal, Instituto Nacional de Investigación y Tecnología Agraria y Alimentaria (INIA-CSIC), Madrid, 28040, Spain

**Keywords:** nulliparous ewes, vaginal microbiota, reproductive management, estrus synchronization, 16S rRNA sequencing

## Abstract

Current knowledge of the ovine vaginal microbiota is limited to adult ewes, leaving the bacterial community of nulliparous ewes and its dynamics during their first reproductive handling undefined. This study aimed to characterize the vaginal microbiota of ewe lambs prior to their first breeding and to evaluate the changes caused by routine handling and estrus synchronization. A total of 66 vaginal samples were collected from 22 nulliparous Latxa ewe lambs at three time points: baseline, post-handling, and post-estrus synchronization using intravaginal devices containing oxytetracycline. Bacterial composition was characterized by sequencing the V3–V4 hypervariable regions of the 16S rRNA gene. Data were processed using DADA2 to infer Amplicon Sequence Variants, and ecological shifts were evaluated through beta and alpha diversity metrics, alongside differential abundance analysis with ANCOM-BC2. A baseline community was revealed, with high diversity and inter-individual variability. This initial state was dominated by the phylum Firmicutes and showed a strong connection to the gut microbiota, including enteric genera such as *Fibrobacter*, *Rikenellaceae* RC9 gut group, and *Lachnospiraceae* NK3A20 group. The first physical examination caused a significant shift in community structure (beta diversity *P* = 0.001), marked by a rapid drop in these gut-associated bacteria and an increase in opportunistic genera such as *Trueperella* (log_2_FC = 2.18; *P*_adj_ < 0.05). The subsequent synchronization treatment further reduced both alpha and beta diversity, creating a uniform environment where genera such as *Aerococcus*, *Corynebacterium*, *Finegoldia*, and *Trueperella* increased, while *Alistipes*, *Acinetobacter*, and *Jeotgalicoccus* decreased (*P*_adj_ < 0.05). Despite these changes, the cohort achieved an 89% pregnancy rate via natural mating. Routine handling and synchronization protocols induce temporary shifts in the vaginal microbiota, decreasing diversity and promoting some opportunistic genera. Crucially, the high fertility rate achieved suggests that these ecological alterations are compatible with successful conception under natural mating conditions.

## Teaser

This study revealed a strong link between vaginal and gut microbiota in nulliparous ewe lambs prior to any manipulation. Although estrus synchronization protocols disrupted this balance, the animals maintained high fertility rates under natural mating.

## Introduction

Sheep production holds substantial economic significance in Spain, positioning the country among the leading producers in Europe and the fifth worldwide. Nevertheless, the sector’s profitability, which is strongly reliant on reproductive performance, continues to represent a major challenge (Ministerio de Agricultura 2022). Despite improvements in reproductive management, fertility rates in ewes remain variable and often suboptimal (Lone 2022; Tadesse et al 2022; Savvulidi et al 2024), partly due to species-specific anatomical and physiological limitations (Masoudi et al 2017). This persistent variability suggests that additional, less-characterized biological factors may be contributing to reproductive outcomes. Within this framework, the reproductive tract microbiota has emerged as a growing area of interest, given its potential role in reproductive physiology.

The relevance of reproductive tract microbiota has been extensively documented in human medicine (Romero et al 2014; MacIntyre et al 2015; Wee et al 2018; Hong et al 2020; Tomaiuolo et al 2020a, 2020b; Bhattacharya et al 2023; Magill and MacDonald 2023) and to a lesser extent in domestic ruminants, with most available studies focused on cattle (Kudo et al 2021; Adnane and Chapwanya 2022; Ault-Seay et al 2023; Webb et al 2023). In ewes, although research remains limited, interest in the vaginal microbiota has grown noticeably in recent years. Within this context, several studies have aimed to characterize its microbial core composition and to identify its modulating factors particularly in adult ewes. Reinoso-Peláez et al (2025a, 2025b) defined a core microbiota using both 16S rRNA metabarcoding and ONT-based metagenomics, identifying the phyla Fusobacteriota, Firmicutes, Proteobacteria, Bacteroidota, and Actinobacteriota as consistently shared across individuals and methods. Similar profiles were also reported in earlier studies by Swartz et al (2014), Serrano et al (2020), and Greenwood et al (2022). At the genus level, *Histophilus*, *Fusobacterium*, *Parvimonas*, *Aerococcus*, *Bacteroides*, and *Streptococcus* were consistently identified as core members (Reinoso-Peláez et al 2025a, 2025b). Other genera, including *Staphylococcus*, *Escherichia*, *Actinobacillus*, *Campylobacter*, *Corynebacterium*, *Klebsiella*, and *Cutibacterium*, have also been reported in ovine vaginal samples (Koester et al 2021; Barba et al 2024; Cassas et al 2024; Reinoso-Peláez et al 2025a, 2025b), although with lower or more variable prevalence.

A growing body of evidence indicates that both genetic and environmental factors shape the composition of the vaginal microbiota in ewes. Breed has emerged as a key genetic determinant, with distinct microbiota profiles reported across ovine breeds (Greenwood et al 2022; Reinoso-Peláez et al 2025a, 2025b). In line with this, Reinoso-Peláez et al (2025c) conducted a microbiota genome-wide association study (mGWAS), identifying numerous host genetic variants associated with the relative abundance of diverse microbial taxa. Environmental factors also play a significant role, as herd-level differences have been consistently observed, often reflecting variations in feeding strategies, reproductive protocols, and geographical conditions (Reinoso-Peláez et al 2025a, 2025b). Age and parity further contribute to microbial shifts, particularly between younger and older ewes (Reinoso-Peláez et al 2025a, 2025b). Similarly, pregnancy status is associated with distinct microbial profiles. For instance, genera such as *Neisseria*, *Oenococcus*, *Mageebacillus*, *Histophilus*, *Actinobacillus*, and *Sneathia* were found more abundant in non-pregnant ewes, whereas *Mannheimia*, *Alistipes*, and members of the *Oscillospiraceae* family were enriched in pregnant animals (Serrano et al 2020; Koester et al 2021; Reinoso-Peláez et al 2023, 2025a, 2025b). Additionally, estrus synchronization protocols using intravaginal pessaries containing progestogen have been shown to alter vaginal microbiota composition (Reinoso-Peláez et al 2023). In fact, previous research has demonstrated that the physical presence of intravaginal devices, combined with exogenous progestogens, alters the normal vaginal bacterial flora, frequently promoting the growth of opportunistic microorganisms (Manes et al 2010; Reinoso-Peláez et al 2023). Collectively, these findings highlight the dynamic interplay between host genetics, physiology, and reproductive management in shaping the vaginal microbial ecosystem.

Although recent studies have expanded our understanding of the vaginal microbiota in ewes, most have concentrated on adult animals, primarily in the context of artificial insemination, pregnancy, or postpartum dynamics (Serrano et al 2020; Koester et al 2021; Greenwood et al 2022; Reinoso-Peláez et al 2023, 2025a, 2025b). Research specifically focused on ewe lambs remains limited. Consequently, little is known about the composition and temporal dynamics of the vaginal microbiota in truly naïve animals, particularly during their first exposure to physical, hormonal, and sexual stimuli. Moreover, differences in breed, management systems, and experimental designs across available studies hinder the extrapolation of findings to other production contexts. Closing this gap is crucial to better understand how the vaginal microbiota is initially established and how it responds to early reproductive events, potentially shaping fertility trajectories in later life.

The current study aims to characterize the composition and temporal dynamics of the vaginal microbiota in nulliparous ewe lambs during their initial reproductive handling. By focusing on the earliest stages of reproductive life, this study aims to 1) characterize the baseline vaginal microbiota in young, reproductively naïve Latxa ewe lambs prior to any intervention; 2) evaluate the microbial shifts following the first intravaginal handling required for estrus synchronization; 3) assess the additional compositional changes induced by intravaginal pessaries containing progestogen immediately before natural mating; and 4) examine the temporal dynamics of taxa previously associated with reproductive outcomes. Through this sequential design, the study offers a detailed view of the microbial transitions linked to early reproductive interventions in ewes.

## Materials and methods

### Animal husbandry

All procedures were minimally invasive and adhered to the Spanish Policy for Animal Protection (RD 53/2013), aligned with the European Union Directive 2010/63/UE. Written informed consent was obtained from the animal owners for participation in this study.

The study involved 22 nulliparous Latxa ewe lambs, with a mean age of 8.2 mo (SD = 0.2) and a mean weight of 41.0 kg (SD = 2.9), raised in a semi-extensive dairy system in Vitoria-Gasteiz, Basque Country, Spain. This system is characterized by high pasture use and a temperate, humid climate. The ewes were in good body condition, had not undergone any prior reproductive procedures, and no clinical signs of disease or infection were observed during the study. The sample size (*n* = 22) was primarily determined by the availability of nulliparous ewes meeting these strict experimental criteria at the farm facility at the time of the study, balanced with logistical and sequencing constraints. To ensure the selected cohort was not closely related and minimize potential confounding effects of host genetics, a genomic relationship matrix (GRM) was constructed following the method of VanRaden (2008) using data from an Illumina high-density 606K SNP chip (mean *r* = −0.039; Figure S1, see online supplementary material for a color version of this figure).

The routine reproductive management began with an initial examination involving the first manual insertion of a sterile speculum to assess vaginal and cervical integrity (Tekin Önder et al 2024). This step, referred to here as initial intravaginal handling, was performed under strict hygienic conditions and resulted in the mechanical rupture of the hymen, a procedural prerequisite to enable the subsequent synchronization procedure. Twelve days later, all ewes were subjected to estrus synchronization using intravaginal sponges containing 20 mg of Flurogestone acetate (Chronogest, MSD Animal Health, Kenilworth, NJ, USA). In accordance with farm protocol, the sponges were dusted with 0.20 mg of oxytetracycline (TENICOL; Merck Sharp & Dohme Animal Health SL, 2083 ESP 02/2024) as an intravaginal antibiotic treatment. After 14 d, the intravaginal sponges were removed and, to synchronize ovulation, each ewe received an intramuscular injection of 400 IU of pregnant mare serum gonadotropin (PMSG). Twenty-four hours after sponge withdrawal, natural mating took place in the same reproductive cycle for all animals (from September 30 to October 5, 2020) using six Latxa adult rams. All rams were in good body condition, with no signs of infection or reproductive disorders. Four ewe lambs were not mated, possibly due to absence of estrus.

### Sampling protocol

**Figure 1:**
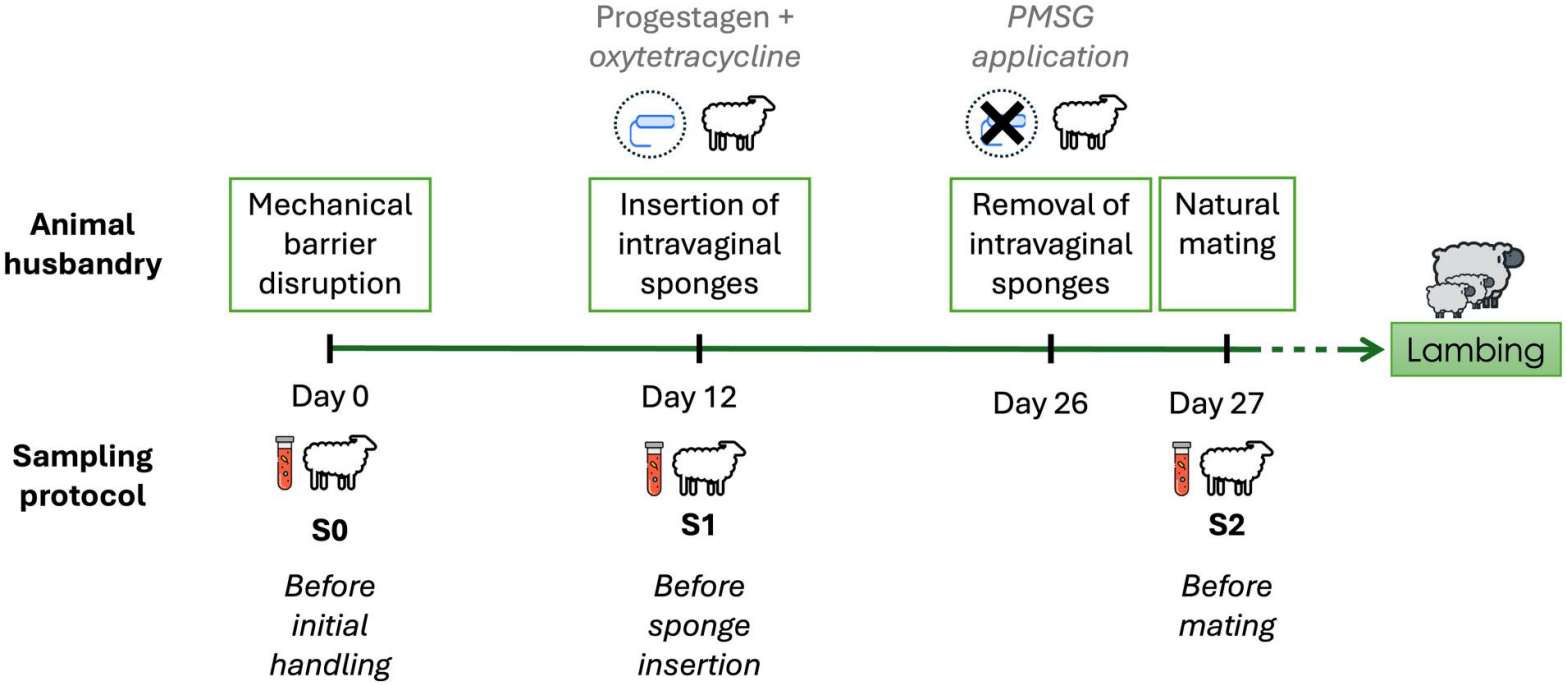
Experimental timeline of animal husbandry interventions and longitudinal sampling protocol. The diagram highlights the three sampling points (S0, S1, and S2). Animal husbandry is shown at the top and the sampling protocol at the bottom. PMSG: pregnant mare serum gonadotropin.

This study involved the collection of one sample from each of the 22 ewes at three specific time points (yielding a total of 66 samples) during the reproductive management described above, conducted by the farm’s veterinarians. To capture the basal state of the vaginal microbiota, the first sample (S0) was collected before any physical intervention, with extreme care taken to preserve the integrity of the hymen. The second vaginal sample (S1) was collected 12 d after the initial speculum examination, immediately prior to the insertion of vaginal sponges, to determine the specific effect of the initial physical disruption. Finally, the third sample (S2) was collected 24 h after the removal of the sponges, just before natural mating took place. See Figure 1 for an illustrative timeline of the animal management and sampling procedures.

All vaginal samples were collected using sterile nylon FLOQSwabs^®^ (Real Vaginal Microbiome DNA Kit, Durviz S.L., Valencia, Spain). Sampling was performed without prior external disinfection to preserve the integrity of the native microbial community. To minimize external contamination, the vulvar folds were manually separated with gloved hands, and the swab was inserted directly into the vaginal mucosa, strictly avoiding contact with the external skin. Following collection, the swab was immediately returned to its original sterile tube. All vaginal samples were stored on dry ice during collection and subsequently frozen at −80°C until DNA extraction.

### DNA extraction

Swabs saturated with vaginal secretions were trimmed and placed into individual Eppendorf tubes. DNA was extracted using the Real Vaginal Microbiome DNA Kit (Durviz S.L., Valencia, Spain) according to the manufacturer’s instructions. DNA samples were eluted in 15 to 30 µL of the elution buffer provided with the kit. Genomic DNA concentration and purity were assessed using both a Qubit 4 fluorometer (Thermo Fisher Scientific, DE, USA) with the Qubit™ dsDNA BR Assay Kit and a Nanodrop 2000 spectrophotometer (Thermo Fisher Scientific, DE, USA), measuring absorbance ratios at 260/280 and 260/230.

### Sequencing

Extracted DNA was used to generate libraries targeting the V3–V4 hypervariable regions of the bacterial 16S ribosomal RNA (rRNA) gene. Amplicon sequencing was performed on an Illumina MiSeq platform (2 × 300 bp paired-end) by an external sequencing service (Instituto de Parasitología y Biomedicina López-Neyra, Granada, Spain). Additionally, a laboratory negative control (PCR blank) was included during library preparation and sequenced alongside the samples to monitor for potential reagent contamination.

### Bioinformatics and statistical analysis

#### Sequence processing and filtering

Amplicons from the V3–V4 hypervariable regions of the 16S rRNA gene were generated using the 341F (5′-CCTACGGGNGGCWGCAG-3′) and 785R (5′-GACTACHVGGGTATCTAATCC-3′) primer pair (Klindworth et al 2013). Primer sequences were trimmed using Cutadapt (Martin 2011). Subsequent sequence processing was performed using the DADA2 package within the R environment (Callahan 2016; R Core Team 2023). Forward and reverse reads were truncated at 240 and 200 bp, respectively, with a maximum of two expected errors, and bases with quality scores ≤2 were trimmed. Amplicon Sequence Variants (ASVs) were inferred and taxonomically classified using the package’s native Naive Bayesian classifier against the SILVA reference database version 132 (https://www.arb-silva.de/download/arb-files/; silva_nr_v132_train_set.fa.gz). All bioinformatic processing was performed using the high-performance computing infrastructure at CESGA (Centro de Supercomputación de Galicia, www.cesga.es). The resulting abundance tables were filtered to exclude ASVs mapping to mitochondrial origin from the host, retaining only sequences taxonomically classified within the Bacteria and Archaea domains.

#### Diversity analysis

To evaluate microbial community structure, beta and alpha diversity were assessed. These analyses were conducted at the ASV level without applying prevalence or abundance filters to preserve the full taxonomic resolution of the dataset and to avoid excluding low-abundance taxa that could contribute to ecologically relevant differences. Beta diversity, defined as the dissimilarity in microbial composition between samples, was assessed using centered log-ratio (CLR) transformation and Euclidean distances with the microbiome R package (Lahti and Shetty 2018). Principal Component Analysis (PCA) was used to visualize patterns of variation across the three sampling points using the prcomp function from the R stats package (R Core Team 2023). Statistical differences in microbial composition were evaluated using Permutational Multivariate Analysis of Variance (PERMANOVA) implemented in the vegan package (Oksanen et al 2022), first under a global model including all sampling points, and subsequently through pairwise comparisons. In both cases, statistical significance was determined using 999 permutations. Subsequently, alpha diversity, defined as microbial diversity within individual samples, was assessed using four commonly applied indices using the Phyloseq package (McMurdie and Holmes 2013): Observed richness, which reflects the number of unique ASVs; Chao1, which estimates ASV richness while accounting for rare taxa; Shannon, which incorporates both richness and evenness; and Inverse Simpson, which measures dominance and evenness within the community. Rarefaction was applied using a depth cutoff of 10,000 reads per sample, as rarefaction curves reached a plateau at this sequencing depth (Figure S2, see online supplementary material for a color version of this figure). Statistical differences in alpha diversity across sampling points were evaluated using the Wilcoxon rank-sum test. All visualization plots were generated with ggplot2 (Wickham 2016).

#### Taxonomic composition

To analyze the microbial core composition, only taxa present in at least 90% of the samples were considered. This prevalence threshold was applied to reduce the influence of sequencing artifacts and extremely low-abundance taxa that could bias compositional analyses. Relative abundances at the phylum and genus levels were then used to describe and visualize the microbial communities shaping the vaginal ecosystem at each time point. To determine if the abundances of these core taxa varied significantly across sampling points, the same statistical framework employed for the differential abundance analysis was applied, as detailed in the corresponding section below.

#### Differential abundance analysis

Finally, differential abundance analysis was performed at the ASV, genus, and phylum levels to identify taxa that shifted significantly between sampling points. This analysis was conducted using the Composition of Microbiomes with Bias Correction (ANCOM-BC2) method (Lin and Das Peddada 2024). Prior to model fitting, a 10% prevalence filter was applied to retain consistent taxa across the dataset. Additionally, group homogeneity was assessed via PERMANOVA (999 permutations) for pregnancy status, age, and body weight; as no significant effects were detected (*P* > 0.05), these variables were not included as covariates in the final models. The ANCOM-BC2 method addresses the compositional nature of microbiome data by explicitly modeling a sample-specific sampling fraction (sequencing bias) within a log-linear regression framework, rather than assuming constant total abundance. To resolve the issue of zero-inflation, a sensitivity analysis regarding pseudo-count addition was employed, ensuring that statistical significance is robust and not an artifact of zero-handling. The model accounted for a repeated-measures design by including Animal ID and Sampling Point as fixed effects, generalized as:


y=Xb+e

where y represents the vector of bias-corrected log-abundances for a given taxon, ***X*** is the incidence matrix corresponding to the fixed effects (Sampling Point and Animal ID), b is the vector of regression coefficients determining the effect size, and e is the vector of residual errors. Pairwise contrasts were performed between consecutive time points (S1 vs S0, S1 vs S2, and S0 vs S2). Significance was determined using Benjamini-Hochberg (FDR) adjusted *P* value (<0.05). Only taxa that were both statistically significant and passed the sensitivity check (pseudo-count addition) were retained and reported as differentially abundant. In addition to the global analysis, a targeted evaluation was conducted focusing on taxa previously reported in the literature as exhibiting differential abundance between pregnant and non-pregnant ewes (Table S1). This literature-based selection allowed assessing whether taxa with established reproductive relevance also displayed significant temporal shifts in the present dataset.

## Results

### Reproductive outcomes

Following the reproductive management and natural mating, the pregnancy rate among the mated ewes was 89%, based on subsequent lambing records.

### Sequencing summary

The sequencing output yielded a total of 897,655 raw reads across the 66 samples. At the ASV level, the largest number of assigned reads was recorded in S1 (375,738), while S0 showed the greatest richness, with 4,103 unique ASVs. At the phylum level, composition remained stable between sampling points. At the genus level, again S1 yielded the highest number of classified reads (318,587) and the greatest number of unique taxa (*n* = 498). No prevalence filter was applied at this stage, since this analysis aimed to explore the overall taxonomic yield prior to downstream filtering. Details are specified in Table 1.

**Table 1:** Summary statistics of read abundance across taxonomic levels.

Level^1^	Group^2^	Total reads^3^	Mean^4^	Median^5^	Number of taxa^6^
**ASV**	S0	278,548	12,661	11,823	4,103
	S1	375,738	17,079	13,312	3,575
	S2	243,369	11,062	9,005	2,709
**Genus**	S0	241,896	10,995	10,270	433
	S1	318,587	14,481	9,944	498
	S2	212,128	9,642	7,762	447
**Phylum**	S0	278,548	12,661	11,823	29
	S1	375,738	17,079	13,312	29
	S2	243,369	11,062	9,005	27

1 Level: Taxonomic classification level.

2 Sampling groups corresponding to the three time points: S0 (before any treatment), S1 (after first intravaginal handling and before vaginal sponge application), and S2 (after vaginal sponge removal and prior to natural mating).

3 All the unclassified reads were discarded at each taxonomic level. No prevalence filters were applied to generate these statistics.

4 The average number of reads per sample.

5 The median number of reads per sample.

6 The total number of distinct taxa identified at each classification level.

### Diversity analyses

**Figure 2:**
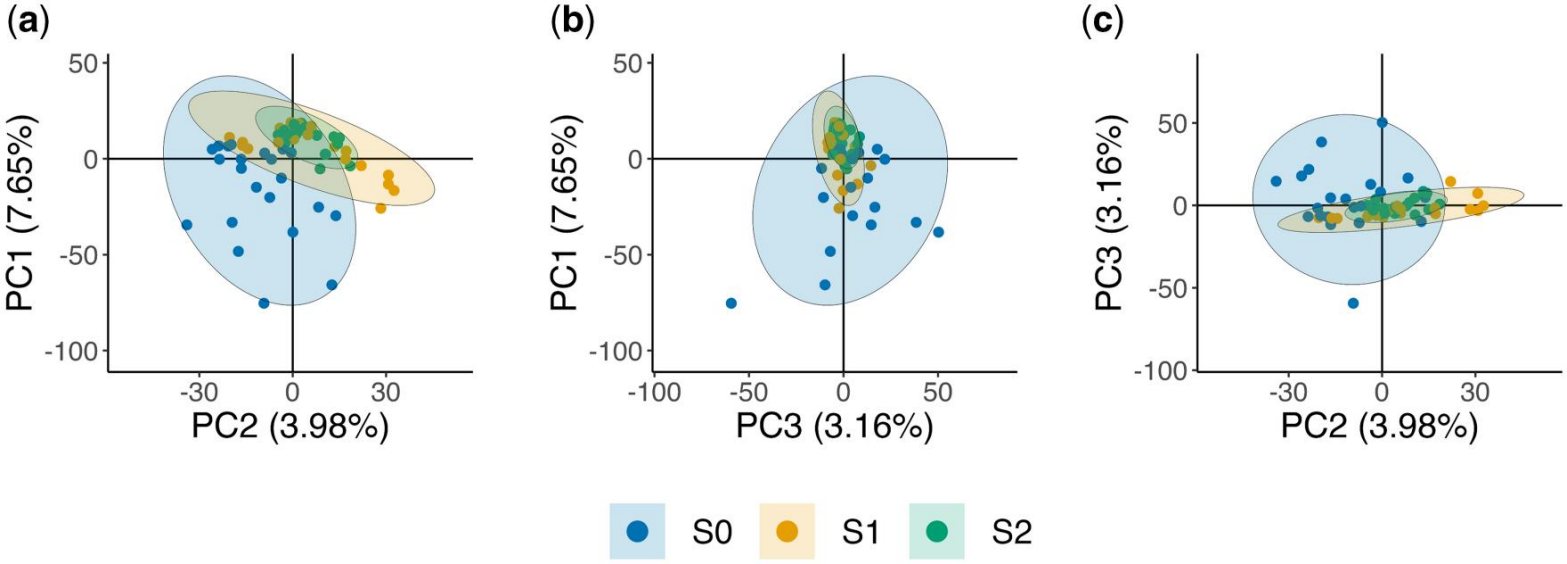
Principal component analysis of vaginal microbiota composition based on ASV-level data across sampling points. Three pairwise combinations of the first three principal components are shown: (**a**) PC1 vs. PC2, (**b**) PC1 vs. PC3, and (**c**) PC2 vs. PC3, with the percentage of explained variance indicated in parentheses. CLR-transformed abundance data were used. Ellipses represent the 95% confidence interval for each group (S0, S1, and S2).

Principal component analysis revealed distinct clustering of S0 samples compared to S1 and S2, particularly in the PC1–PC2 plane, while some overlap was observed between S1 and S2 (Figure 2). PC1 explained 7.65% of the total variance, followed by PC2 (3.98%) and PC3 (3.16%). Results from PERMANOVA revealed significant differences in vaginal microbial composition across the three sampling points at ASV level (*F* = 2.06, *R*^2^ = 0.061, *P* = 0.001). Pairwise comparisons confirmed that all sampling groups differed significantly from each other (S0 vs S1, S0 vs S2, and S1 vs S2; all *P* = 0.001, FDR-adjusted).

**Figure 3:**
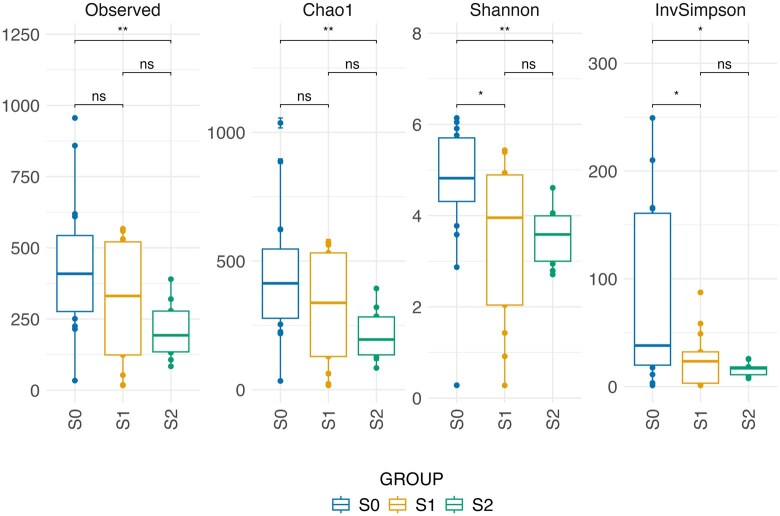
Alpha diversity metrics across sampling points. Alpha diversity was calculated at the ASV level using rarefied data (10,000 reads/sample). Each boxplot represents the distribution of diversity values for the three sampling groups (S0, S1, S2). Statistical comparisons between time points were assessed using the Wilcoxon rank-sum test. Significance levels are denoted as follows: *P < 0.05, **P < 0.01, and “ns” indicates no significant difference.

Alpha diversity significantly varied across sampling points (Figure 3). Richness indices (Observed and Chao1) were highest in S0 and progressively decreased in S1 and S2, although statistically significant differences were only detected between S0 and S2. The same trend was observed for diversity metrics accounting for evenness (Shannon and Inverse Simpson), showing in this case significant reductions between S0 and S1, with no further significant changes between S1 and S2. Overall, these results indicate a progressive decline in richness over time.

### Taxonomic composition

**Figure 4:**
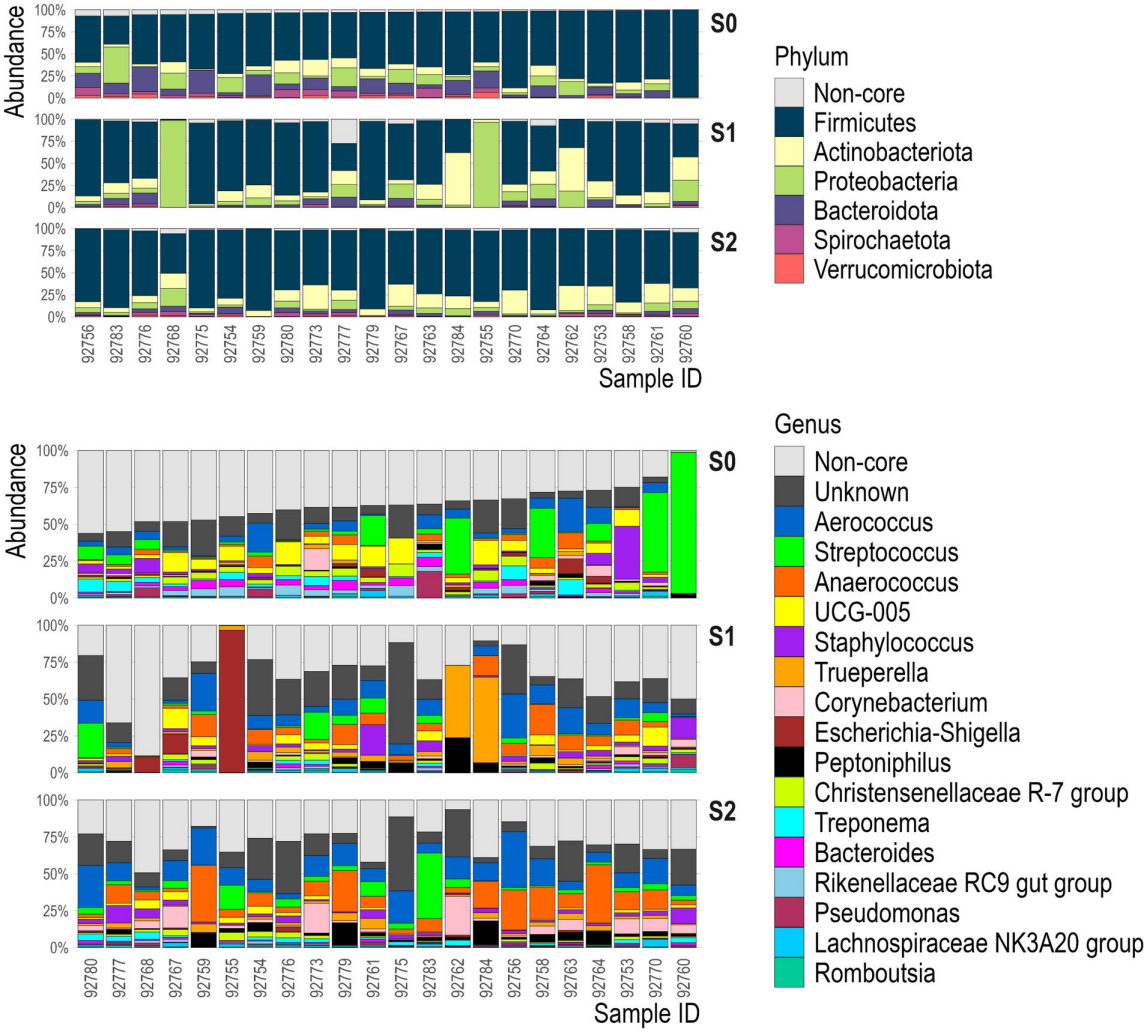
Microbial core composition at the phylum and genus levels across the three sampling points. Only taxa with a prevalence ≥90% were retained for visualization. The 15 most abundant taxa are shown; all others were grouped under the category “Others.” ASVs with no taxonomic assignment in the reference database are labeled as “Unknown.” Sample order on the *x*-axis was determined by the most abundant taxonomic group in S0, specifically Firmicutes at the phylum level and “Others” at the genus level.

At the phylum level, the most abundant taxa across all samples were Firmicutes (66.4%), Actinobacteriota (11.7%), Proteobacteria (10.2%), Bacteroidota (6.1%), and Spirochaetota (1.9%). Firmicutes was predominant in all groups, particularly in S2 (73.4%). Actinobacteriota and Proteobacteria were more abundant in S1 (14.4% and 15.7%, respectively), while Bacteroidota and Spirochaetota reached their highest value in S0 (11.3% and 3.3%). At the genus level, the most abundant taxa were *Aerococcus* (9.7%), *Streptococcus* (7.7%), *Anaerococcus* (7.5%), *Oscillospiraceae* UCG-005 (4.2%), and *Staphylococcus* (3.6%). *Aerococcus* and *Anaerococcus* increased notably in S2 (13.9% and 13.1%, respectively), whereas *Streptococcus* was more abundant in S0 (13.4%). *Oscillospiraceae* UCG-005 was highest in S0 (7.2%) and decreased progressively through S1 and S2. *Staphylococcus* showed relatively stable abundances across the three sampling points. Full details at the phylum and genus levels are shown in Figure 4 and Table 2.

**Table 2:** Relative abundance of the most prevalent genera and phyla across the three sampling points.

	S0^2^	S1	S2	Mean^3^
**Phylum** ^1^				
**Firmicutes**	64.60^a^	61.00^a^	73.40^a^	66.33
**Actinobacteriota**	6.69^a^	14.4^a^	14.0^a^	11.69
**Proteobacteria**	9.45^a^	15.7^a^	5.14^a^	10.11
**Bacteroidota**	**11.2^a,b^**	**4.08^a^**	**3.05^b^**	6.13
**Spirochaetota**	**3.32^a^**	**0.63^b^**	**1.79^a,b^**	1.91
**Verrucomicrobiota**	1.64^a^	0.42^a^	0.80^a^	0.96
**Genus**				
***Aerococcus***	5.55^a^	9.26^a^	13.7^a^	9.49
***Streptococcus***	13.3^a^	3.8^a^	5.72^a^	7.61
***Anaerococcus***	**2.82^a,b^**	**6.43^a^**	**12.9^b^**	7.40
***Oscillospiraceae* UCG-005**	**7.08^a,b^**	**3.4^a^**	**1.81^b^**	4.10
***Staphylococcus***	3.66^a^	3.58^a^	3.3^a^	3.51
***Trueperella***	**0.665^a^**	**7.18^b^**	**2.42^a^**	3.42
***Corynebacterium***	**1.77^a,b^**	**1.34^a^**	**5.54^b^**	2.88
***Escherichia-Shigella***	1.57^a^	5.72^a^	0.559^a^	2.61
***Peptoniphilus***	**0.826^a,b^**	**2.73^a^**	**4.02^b^**	2.53
***Christensenellaceae* R-7 group**	3.49^a^	1.65^a^	1.54^a^	2.23
***Treponema***	3.05^a^	0.628^a^	1.79^a^	1.82
***Bacteroides***	**2.7^a^**	**0.75^b^**	**0.815^a,b^**	1.42
***Rikenellaceae* RC9 gut group**	**2.81^a^**	**0.636^b^**	**0.505^a^**	1.32
***Pseudomonas***	1.87^a^	0.854^a^	0.65^a^	1.13
***Lachnospiraceae* NK3A20 group**	1.02^a^	0.943^a^	0.961^a^	0.98
***Romboutsia***	0.415^a^	0.595^a^	0.421^a^	0.48

1 Only taxa with a prevalence ≥90% across the dataset were retained; the 15 most abundant genera and phyla are shown, and all remaining taxa were grouped under “Other.” Taxa with no classification in the reference database are labeled “Unknown.”

2 Values in bold indicate sampling groups in which at least one significant change was detected.

3 Relative abundance averaged across all samples.

^a,b^Within each row, sampling groups (S0, S1, S2) that share a letter do not differ significantly, whereas groups with no letter in common differ significantly (ANCOM-BC2 pairwise comparisons; Benjamini–Hochberg-adjusted *P* < 0.05, verified by sensitivity analysis to pseudo-count imputation); “a,b” denotes a group that differs from neither “a” nor “b.”

Within the core microbiota, several significant changes were observed. From S0 to S1 (ANCOM-BC2; FDR < 0.05 with robust sensitivity check), at the phylum level, Spirochaetota decreased. At the genus level, *Trueperella* increased, whereas *Bacteroides* and the *Rikenellaceae* RC9 gut group decreased. From S1 to S2, the only significant change at the phylum level was a decrease in Bacteroidota, while at the genus level, *Anaerococcus*, *Corynebacterium*, and *Peptoniphilus* increased, concurrent with decreases in *Trueperella* and the *Rikenellaceae* RC9 gut group (Table 2).

### Differential abundance analysis

**Figure 5:**
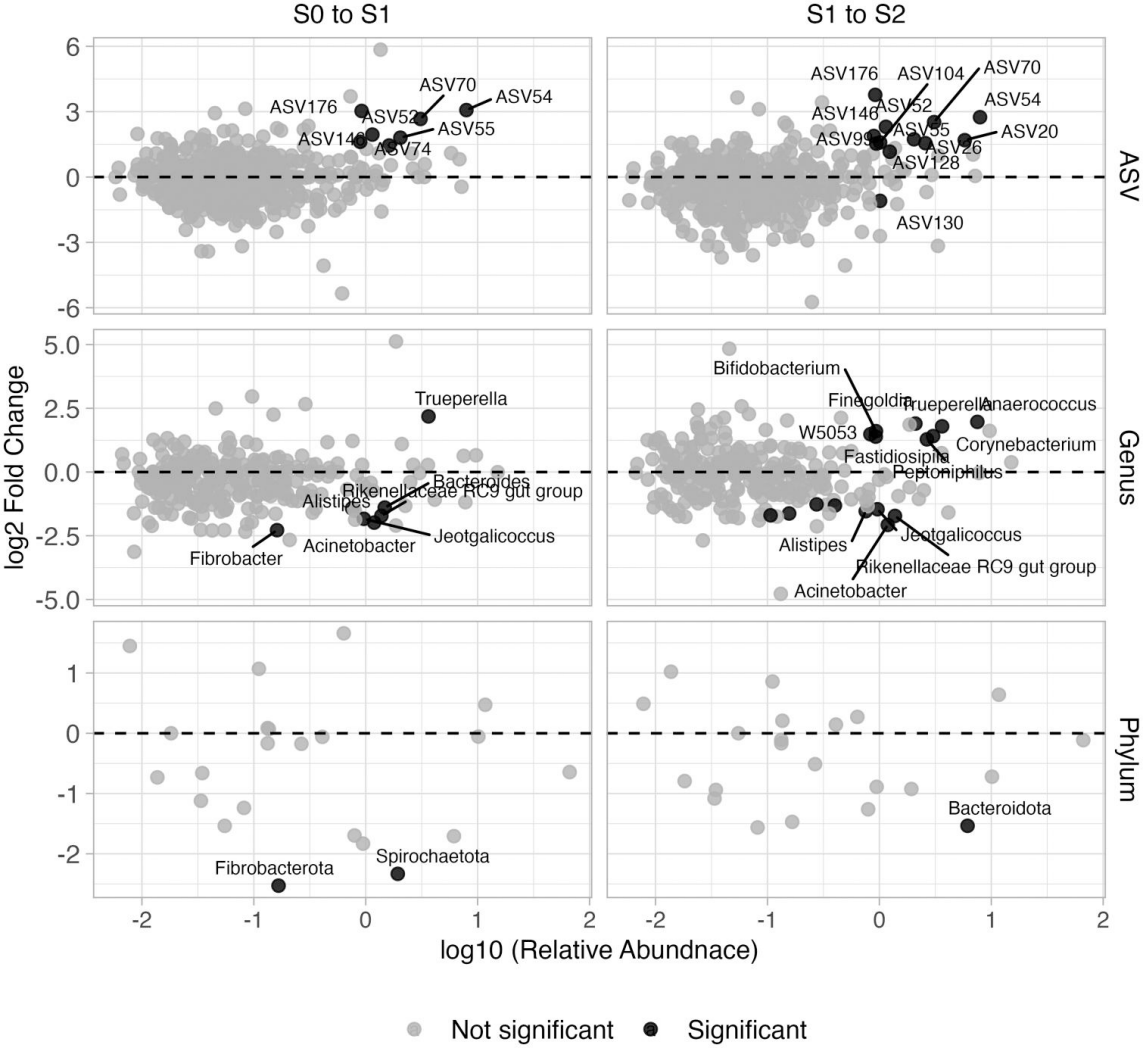
MA plots of differential abundance analysis across sampling points. The plots display log_2_FC (y-axis) against the log10 mean relative abundance (x-axis) for microbial taxa, derived from the ANCOM-BC2 analysis. Each panel shows a pairwise comparison: “S0 to S1” or “S1 to S2,” at the ASV, genus, and phylum levels. In the “S0 to S1” plots, positive log_2_FC values indicate taxa more abundant in S1, while negative values indicate higher abundance in S0. Similarly, in the “S1 to S2” plots, positive values correspond to taxa enriched in S2, and negative values to those more abundant in S1. The x-axis reflects the mean relative abundance on a log10 scale; the value of 0 corresponds to 1% mean abundance. Negative values represent taxa below 1% (−1 is 0.1% and −2 is 0.01%), and positive values represent taxa above 1% (1 is 10% and 2 is 100%). Only taxa with a prevalence ≥10% were considered. Significant taxa (*P*_adj_ < 0.05) are colored in black; non-significant taxa are shown in gray.

Overall, the highest number of significant taxa occurred at the ASV and genus levels (Figure 5, Table 3). In the S0–S1 comparison, seven ASVs, seven genera, and two phyla were significantly different. In contrast, the S1–S2 comparison identified 12 ASVs, 16 genera, and 1 phylum showing shifts in abundance. In the S0–S2 comparison, only one genus (*Bifidobacterium*, log2FC = 2.06; Mean RA = 0.43) was identified as differentially abundant.

**Table 3:** Differential abundance of microbial taxa across sampling points in ewe lambs.

Level	Taxa	S0 to S1^1^	S1 to S2	Mean RA, %^2^
**Phylum**	Fibrobacterota	−2.53		0.17
	Spirochaetota	−2.33		1.93
	Bacteroidota		−1.54	6.14
**Genus**	*Trueperella*	2.18	1.79	3.64
	*Bacteroides*	−1.39		1.48
	*Rikenellaceae* RC9 gut group	−1.72	−1.72	1.38
	*Fibrobacter*	−2.28		0.16
	*Alistipes*	−1.43	−1.54	0.76
	*Jeotgalicoccus*	−1.84	−1.46	0.96
	*Acinetobacter*	−1.99	−2.08	1.19
	*Bifidobacterium*		1.6	0.93
	*Prevotellaceae* UCG-003		−1.63	0.16
	*Corynebacterium*		1.41	3.02
	*Finegoldia*		1.9	2.1
	*Aerococcus*		1.61	9.66
	[*Ruminococcus*] *torques* group		−1.27	0.27
	*Lachnospiraceae* AC2044 group		−1.31	0.4
	*Lachnospiraceae* UCG-001		−1.7	0.11
	*Fastidiosipila*		1.39	0.93
	*Anaerococcus*		1.97	7.51
	*Peptoniphilus*		1.28	2.66
	W5053		1.48	0.83
**ASV**	ASV74 (*Trueperella*)	1.45		1.62
	ASV146 (*Trueperella*)	1.62	1.88	0.89
	ASV104 (*Finegoldia*)		1.59	1.02
	ASV128 (*Corynebacterium*)		1.16	1.24
	ASV52 (Firmicutes/NA)	1.95	2.31	1.15
	ASV54 (Firmicutes/NA)	3.07	2.75	7.93
	ASV70 (Firmicutes/NA)	2.66	2.52	3.09
	ASV176 (Firmicutes/NA)	3.03	3.77	0.92
	ASV20 (*Anaerococcus*)		1.69	5.8
	ASV26 (*Anaerococcus*)		1.56	2.58
	ASV99 (W5053)		1.54	0.94
	ASV130 (UCG-005)		−1.09	1.02

1 Results are derived from ANCOM-BC2 analysis; only taxa meeting the dual criterion of statistical significance (Benjamini–Hochberg-adjusted *P* < 0.05) and a successful sensitivity check for pseudo-count addition are reported. Values are log2FC: positive values indicate higher abundance at the later time point relative to the earlier one, and negative values indicate lower abundance. Empty cells denote comparisons that were not significant or did not pass the robust sensitivity check.

2 Global mean relative abundance (%) of each taxon across the dataset.

**Figure 6:**
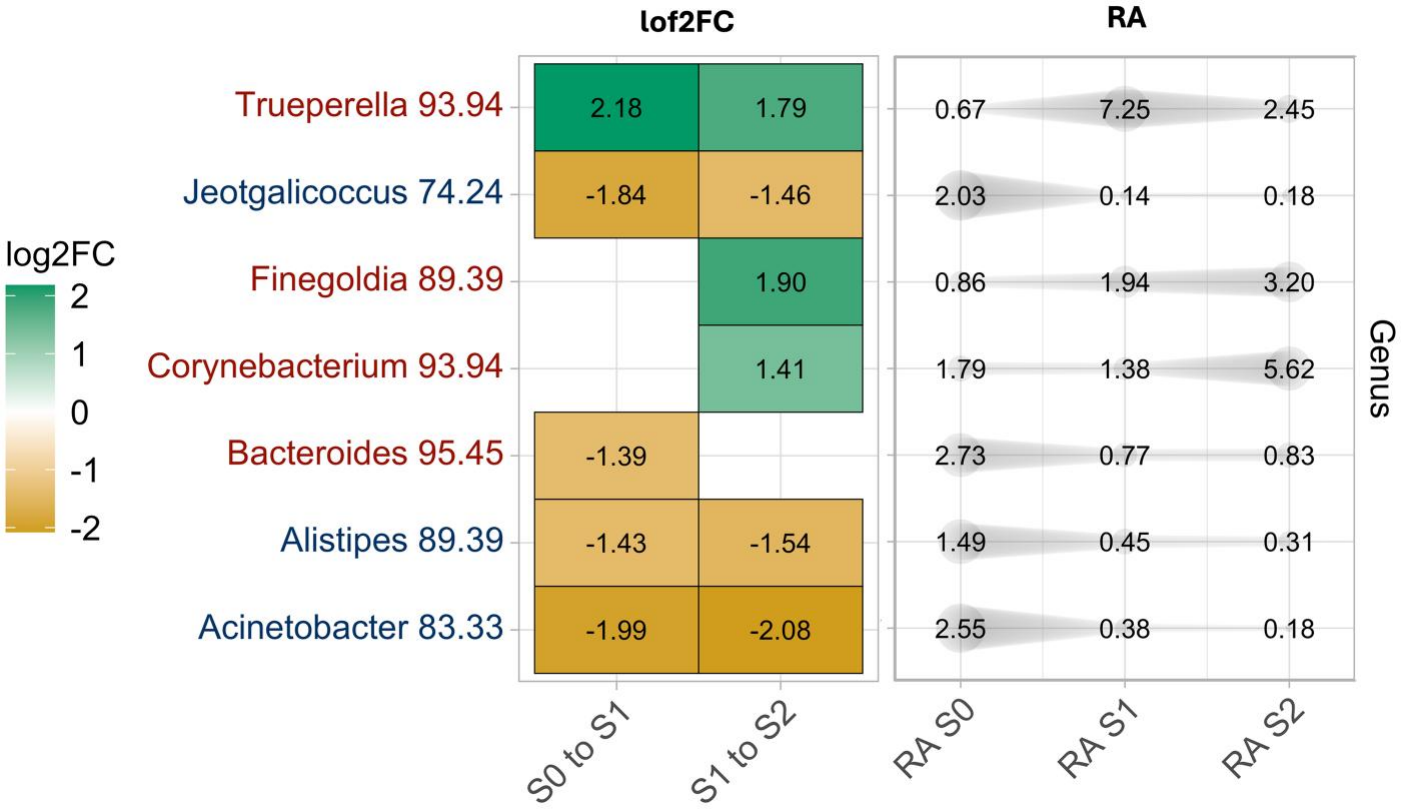
Heatmap of significant taxa (*P*_adj_ < 0.05) previously linked to pregnancy status in ewes. The y-axis lists the taxa according to their previously reported association: more abundant in non-pregnant ewes (Trueperella, Finegoldia, Corynebacterium, and Bacteroides) or more abundant in pregnant ewes (Jeotgalicoccus, Alistipes, and Acinetobacter). The left columns display the log_2_FC for each pairwise comparison (S0 to S1 and S1 to S2): for S0–S1, positive values indicate taxa more abundant in S1 and negative values taxa more abundant in S0; for S1–S2, positive values indicate higher abundance in S2 and negative values higher abundance in S1. The log2FC value is displayed within each cell, with positive values indicating an increase and negative values a decrease.The right-hand columns show the mean relative abundances (RA) for each taxon in S0, S1, and S2, represented as bands whose vertical thickness corresponds to the abundance.

Among the taxa showing significant changes, the analysis focused on those previously reported in the literature as exhibiting differential abundance based on pregnancy status (Figure 6). Genera associated with non-pregnant ewes, such as *Finegoldia* and *Corynebacterium*, significantly increased from S1 to S2. In contrast, *Bacteroides* decreased from S0 to S1 and remained low in S2. Conversely, genera reported as more abundant in pregnant ewes, including *Jeotgalicoccus*, *Alistipes*, and *Acinetobacter*, exhibited a progressive decline, with significant reductions observed from S0 to S1, and continuing from S1 to S2.

These patterns were confirmed at the ASV level. The initial increase in *Trueperella* was mainly due to ASV74 and ASV146. Later, the rise in *Finegoldia* and *Corynebacterium* was linked to the specific appearance of ASV104 and ASV128, respectively (see Table 3).

## Discussion

In this study we have characterized the vaginal microbiota of nulliparous ewe lambs during their initial reproductive management. Unlike adult ewes, these animals are reproductively naïve, providing a unique baseline to observe the ecological impact of standard synchronization protocols and reproductive management. Our data reveal that the process of hymen removal is followed by a rapid shift in microbial composition, challenging the assumption that such procedures are biologically neutral.

### Baseline composition

At baseline (S0), the vaginal community exhibited high taxonomic richness alongside high inter-individual variability, especially at genus level. This heterogeneity likely stems from two biological factors. First, the S0 group was not synchronized, and it is known that differences in the natural estrus cycle create distinct hormonal pressures on the microbiota composition (Swartz 2014; Koester et al 2021; Barba et al 2024). Second, the nulliparous status implies a lack of prior reproductive activity (e.g., synchronization, mating, or parturition), which typically acts as a selective force to unify the microbiota (Barba et al 2024; Reinoso-Peláez et al 2023, 2025b). Consequently, the baseline ecosystem appears to be shaped by stochastic environmental colonization rather than a uniform, adapted profile.

Taxonomically, the co-dominance of Firmicutes, Bacteroidota, Proteobacteria, and Actinobacteriota aligns with the nulliparous cohort described by Barba et al (2024). However, at the genus level, the dominance hierarchy differed. Whereas Barba et al (2024) reported a predominance of *Porphyromonas*, our data indicate a community mainly driven by *Streptococcus, Oscillospiraceae* UCG-005, and *Aerococcus*. These taxonomic variations likely reflect the combined influence of host genetics (Latxa vs. Churra breeds), as well as environmental and management differences between the Basque Country and Castilla y León.

Notably, S0 composition revealed a distinct “gut-associated” signature, characterized by enteric taxa such as *Fibrobacter*, *Oscillospiraceae* UCG-005, *Christensenellaceae* R-7 group, *Rikenellaceae* RC9 gut group, *Escherichia-Shigella*, and *Bacteroides* (Liang et al 2022; Jiang et al 2023; Shin et al 2024). This ecological overlap is likely driven by anatomical proximity and potential microbial migration between the rectum and the reproductive tract (Escorcia Mora et al 2025; Takada 2025). Consequently, these typically intestinal genera may persist as integral components of the vaginal community rather than mere transient contaminants. Indeed, genera like *Escherichia* and *Bacteroides* carry enzymes such as beta-glucuronidase, which can deconjugate estrogens and modulate their reabsorption and bioavailability (Lander et al 2001; Kwa et al 2016). This functional capacity implies that the gut-associated bacteria identified in our samples may play an active role in regulating the local hormonal milieu prior to the onset of the reproductive cycle.

Comparisons with previously reported adult ewe cohorts further highlight the potential effect of life stage on microbiota structure. While Serrano et al (2020), Koester et al (2021), and Reinoso-Peláez et al (2025b) reported Proteobacteria levels of 16–20% in adult flocks, the nulliparous ewes in this study displayed markedly lower abundance (∼10%). Instead, our cohort was largely dominated by Firmicutes (66%). At the genus level, research on adult ewes frequently identifies *Histophilus*, *Fusobacterium*, *Ureaplasma*, and *Parvimonas* as core or dominant taxa (Serrano et al 2020; Koester et al 2021; Reinoso-Peláez et al 2025a, 2025b). Conversely, these genera were absent or detected at negligible levels in our nulliparous cohort. This divergence likely reflects the reproductive immaturity of the ewe lambs, which have not yet undergone mating or lambing events, which facilitate microbial transfer and enrichment of host-associated pathobionts (Serrano et al 2020; Barba et al 2024).

### Impact of initial handling (hymen rupture; S0 to S1)

The first intravaginal manipulation was associated with an immediate restructuring of the bacterial community. This physical intervention was followed by a reduction of alpha diversity and a shift in community structure, as evidenced by the reduction of variability from S0 to S1 in the PCAs. This decline in richness suggests that the initial handling acts as an environmental filter, eliminating less adapted microorganisms and favoring a more restricted set of taxa.

A defining feature of this transition was the significant depletion of enteric taxa. Genera such as *Bacteroides and Rikenellaceae* RC9 gut group, and *Fibrobacter* experienced a decrease in S1 relative to S0. As hypothesized in the baseline analysis, these groups likely represent exogenous bacteria derived from the gut environment. Their inability to persist following a physical disturbance supports the notion that they are not true colonizers of the vaginal mucosa. The physical cleaning or the mechanical stress of the speculum insertion appears to disrupt their temporary adherence, leading to their rapid clearance.

Concomitant with the loss of diversity, *Trueperella* exhibited the most significant increase, with a log2FC of 2.18. This genus, particularly *T. pyogenes*, is a well-known opportunistic pathogen in ruminants, frequently associated with suppurative infections (Bicalho et al 2016; Rzewuska et al 2019). Its sudden proliferation suggests that the handling procedure may create favorable conditions for its expansion. This shift was accompanied by a marked expansion of unclassified Firmicutes variants (ASVs 54, 70, 176), suggesting a broader restructuring of the community toward specific fermentative taxa. Two mechanisms could explain this shift. First, the hymen removal might cause mucosal micro-trauma, exposing extracellular matrix components that facilitate *Trueperella* adhesion. Second, the opening of the vaginal canal introduces atmospheric oxygen. Since *Trueperella* is a facultative anaerobe (Rzewuska et al 2019), it may hold a competitive advantage over the strictly anaerobic obligates that dominate the undisturbed niche. Additionally, genera such as *Jeotgalicoccus*, *Acinetobacter*, and *Alistipes* which have been reported as more abundant in pregnant ewes, decreased significantly in S1 (Koester et al 2021; Reinoso-Peláez et al 2025a). The reduction of these groups, alongside the rise of opportunistic pathogens like *Trueperella*, indicates that the hymen removal drives the microbiota toward a state of temporary dysbiosis. This transition challenges the neutrality of routine reproductive examinations, highlighting that even standard procedures can destabilize the commensal microflora before hormonal synchronization begins.

### Impact of estrus synchronization (S1 to S2)

The insertion of vaginal sponges, that contain antibiotics, and potential host immune responses to the foreign body, exerted a selective pressure on the vaginal ecosystem. Following this intervention, the community shifted toward its lowest state of diversity. As observed in the alpha diversity metrics, which reached their minimum values in S2, and further reflected in the PCA, where S2 samples showed the lowest variance between samples, indicating that the combination of hormonal priming and antibiotic exposure creates a highly uniform environment.

A critical finding in this phase is the alignment of the S2 microbiota with profiles previously associated with reproductive failure (Smith et al 2016; Serrano et al 2020; Koester et al 2021; Reinoso-Peláez et al 2025a, 2025b). Taxa previously associated with higher abundance in non-pregnant ewes, particularly *Trueperella* (already elevated in S1), *Finegoldia*, and *Corynebacterium*, showed a significant increase during the synchronization period. The expansion of these genera suggests a specific resistance to oxytetracycline or an ability to thrive in the anaerobic, progesterone-rich microenvironment created by the sponge. Conversely, genera associated to be more abundant in pregnant ewes, such as *Jeotgalicoccus*, *Acinetobacter*, and *Alistipes*, continued their decline.

In this way, the ewes achieved the mating phase with a bacterial profile that appeared “unfavorable” for conception. However, despite the rise of opportunistic taxa and the loss of beneficial bacteria, the ewes achieved a pregnancy rate of 89%. Several biological factors may explain this outcome. Crucially, reproduction was performed via natural mating. In ovine production systems, natural service consistently yields higher fertility rates compared to artificial insemination, providing a biological advantage that may override the potential negative effects of the observed dysbiosis. Additionally, the timing of sample collection is a key factor. Samples were obtained 24 h after sponges removal, reflecting the specific microenvironment generated by the exogenous progestogens and vaginal devices. It is well-documented in ewes that high progesterone concentrations down-regulate uterine immune functions, increasing susceptibility to bacterial persistence (Hansen 1998). However, the subsequent removal of the device triggers the follicular phase and a surge in circulating estrogens. This hormonal shift upregulates local immune defense mechanisms, inducing mucus hypersecretion and neutrophil recruitment that effectively clear bacterial contaminants (Lewis 2003). These physiological mechanisms likely explain why temporary dysbiosis does not necessarily impede fertility. These observations align with Manes et al (2010) and Reinoso-Peláez et al (2023), who reported microbiological shifts after removing intravaginal devices, including an increase in opportunistic bacteria, but concluded that despite these significant alterations in the local flora, the subsequent pregnancy rates remained unaffected, highlighting the resilience of the reproductive tract. Extending longitudinal monitoring into early gestation is advisable to define the precise timeline of microbial restoration and to elucidate the functional interactions between the recovering ecosystem and the host immune response during the critical window of implantation.

Furthermore, it is important to interpret with caution the pathogenicity of the identified bacteria. The ASVs detected in this study might not correspond to the highly virulent strains typically associated with clinical metritis or abortion. It is also relevant to note that *Trueperella*, *Finegoldia*, and *Corynebacterium* are members of the microbial core. Previous studies have associated these bacteria with reproductive outcomes based on their relative abundance rather than their simple presence or absence (Smith et al 2016; Reinoso-Peláez et al 2025a, 2025b). Therefore, the microbial shift observed here may represent a state of temporary dysbiosis rather than a pathogenic infection. This suggests that the vaginal niche can recover its functionality and support fertilization even after significant ecological restructuring.

### Study limitations

While this study provides valuable insights into the vaginal microbiota dynamics of ewe lambs, some methodological considerations should be noted. First, the study design lacked an untreated control group. Because all animals underwent the same reproductive interventions simultaneously, it is challenging to fully separate the specific effects of physical handling and hormonal synchronization from natural temporal variations or host maturation. Consequently, the observed microbial shifts are temporally associated with the management procedures, but absolute causality cannot be established. Second, the sample size included in this study (*n* = 22) is relatively limited, which may constrain the statistical power and broad generalizability of the findings. Nevertheless, the longitudinal repeated-measures design employed here partly compensates for this limitation by reducing inter-individual confounding factors and enhancing the power to detect true intra-individual temporal shifts. Third, baseline comparisons between nulliparous ewe lambs and adult ewes were contextualized using existing literature, as a contemporaneous adult cohort was not included in our experimental design. Finally, regarding sampling protocols, although strict hygienic measures were followed, environmental field swabs were not collected, which limits the ability to completely evaluate potential background environmental DNA from the farm.

## Conclusions

This study provides the first characterization of the vaginal microbiota in nulliparous Latxa ewe lambs and evaluates its response to standard reproductive management. The vaginal ecosystem of these reproductively nulliparous ewes is characterized by high taxonomic richness and inter-individual variability. Unlike adult ewes, this niche is dominated by Firmicutes and exhibits a strong “gut-associated” signature, marked by the presence of transient enteric taxa such as *Bacteroides* and *Fibrobacter*, likely due to anatomical proximity and the absence of prior reproductive filtering. The initial intravaginal handling (hymen removal) acts as an immediate environmental filter. This physical intervention may interfere with the adherence of exogenous enteric bacteria and is associated with a rapid increase in opportunistic pathogens such as *Trueperella*. These findings indicate that routine reproductive examinations are not biologically neutral and are associated with an early state of dysbiosis. Furthermore, the application of vaginal sponges with antibiotics drives the community toward its lowest diversity and establishes a uniform, treatment-adapted environment. This selective pressure favors fermentative genera like *Corynebacterium* and *Finegoldia* while reducing the relative abundance of taxa previously reported as beneficial for pregnancy, such as *Jeotgalicoccus*, *Alistipes*, and *Acinetobacter*. Consequently, prior to mating, the ewes presented a microbiological profile theoretically “unfavorable” for conception. However, the high pregnancy rate (89%) suggests that this dysbiosis is transient. Physiological mechanisms activated during estrus, particularly estrogen-driven mucosal clearance, likely restore ecological balance and tissue functionality.

## Supplementary Material

skag184_Supplementary_Data

## Data Availability

Sequence data that support the findings of this study have been deposited in the Zenodo repository with the DOI: https://doi.org/10.5281/zenodo.18335569.

## Abbreviations

ASV: amplicon sequence variant
CLR: centered log-ratio
FC: fold-change
FDR: false discovery rate
GRM: genomic relationship matrix
log2FC: log2 fold-change
mGWAS: microbiota genome-wide association study
ONT: Oxford Nanopore Technologies
PCA: principal component analysis
PERMANOVA: permutational multivariate analysis of variance
PMSG: pregnant mare serum gonadotropin
RA: relative abundance
rRNA: ribosomal RNA
SNP: single nucleotide polymorphism
