# Supplementary material for: Shifts in the vaginal microbiota of nulliparous ewe lambs following initial reproductive handling and estrus synchronization
Source: J Anim Sci. 2026 Jun 5;104:skag184. doi: 10.1093/jas/skag184 (PMC13344839; doi:10.1093/jas/skag184)
Supplement: skag184_Supplementary_Data [file skag184_supplementary_data.pdf]

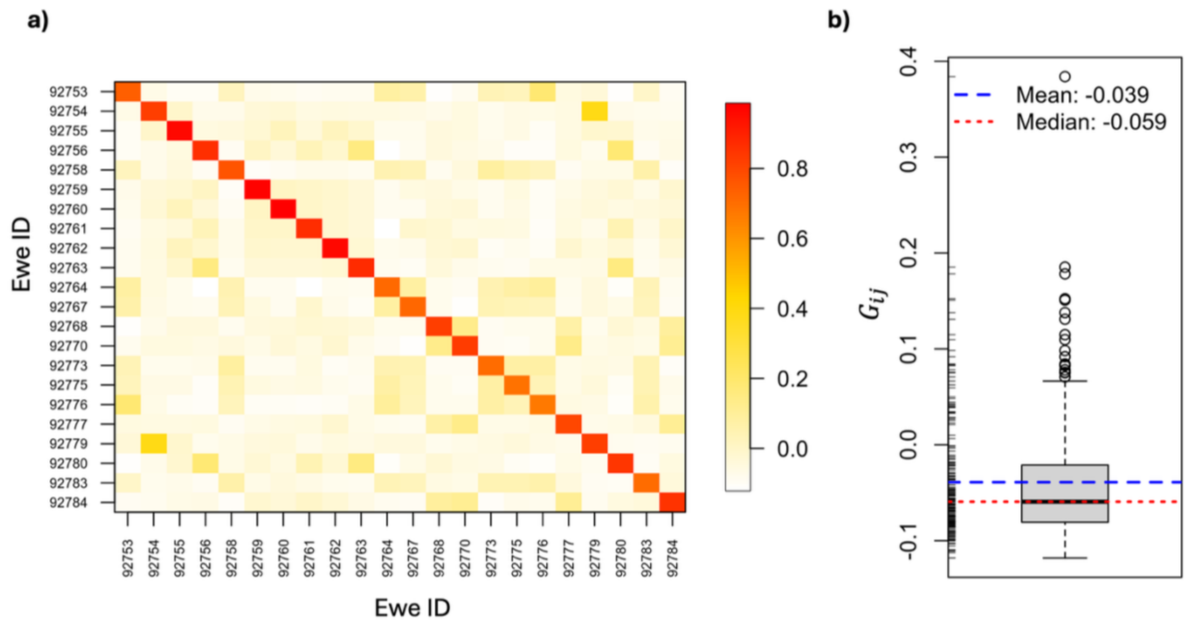

Figure S1. Genomic relationship matrix and distribution of genomic relationship values. Panel (**a**) shows the genomic relationship matrix for 22 ewe lambs (VanRaden 2008 method). Rows and columns correspond to Ewe lamb's ID. The colour scale represents the genomic relationship value G_{ij}. Diagonal cells G_{ii} represent each animal with itself. Panel (b) shows a boxplot of the G_{ij} values. Dashed lines indicate the sample mean (blue) and the sample median (red).

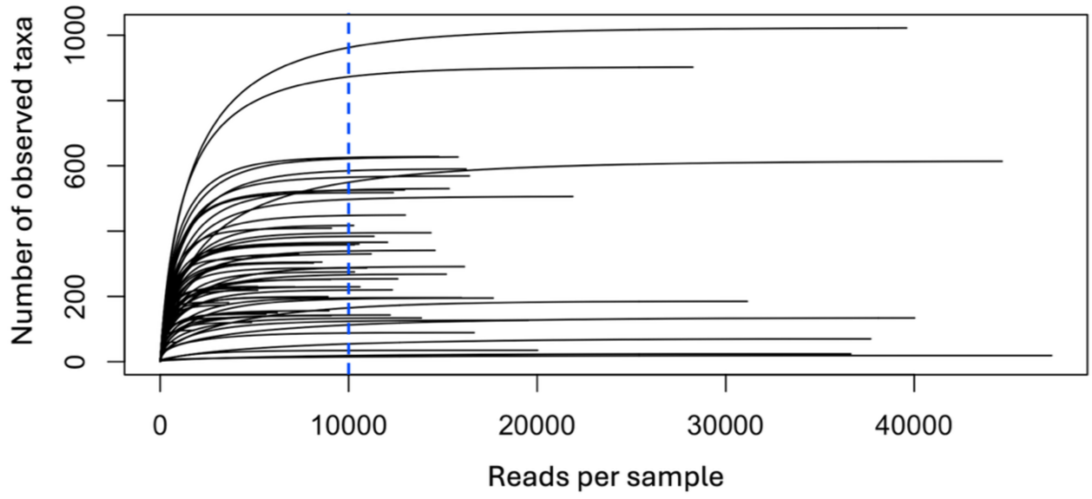

Figure S2. Rarefaction curves of observed ASVs per sample. Each line corresponds to one sample; the x-axis shows sequencing depth (reads per sample) and the y-axis the number of observed ASVs. The blue dashed vertical line marks the rarefaction depth used for downstream analyses (10,000 reads per sample).

Table S1. Literature-derived taxa reported as differentially abundant between pregnant and non-pregnant ewes

Level	High abundant	Taxa
Phylum	Non-pregnant	Fusobacteriota [S1, S2]
Genus	Pregnant	Acinetobacter [S1], Actinobacillus [S4], Aerococcus [S1], Alistipes [S3], Alloicoccus [S4], Arcanobacterium [S1], Atopostipes [S4], Bacteroides [S3], Brevundimonas [S1], Fluviicola [S5], Glutamicibacter [S3], Jeotgalicoccus [S3], Lactobacillus [S6], Mannheimia [S3], Methanobrevibacter [S3], Methanosphaera [S3], Mogibacterium [S3], Phascolarctobacterium [S3], Pseudomonas [S1], Romboutsia [S3], Streptococcus [S3], Streptomyces [S5], Turicibacter [S3], Ureaplasma [S4]
	Non-pregnant	Acinetobacter [S3], Actinobacillus [S5], Anaplasma [S7], Bacteroides [S1, S2], Bergeyella [S3], Brevibacterium [S3], Brucella [S8], Campylobacter [S2, S8, S9], Chlamydia [S8], Corynebacterium [S3, S5], Coxiella [S7, S10], Erysipelothrix [S5], Escherichia [S1, S5], Facklamia [S3], Falsiporphyromonas [S3], Finegoldia [S11], Flavobacterium [S3], Fusobacterium [S1, S3], Histophilus [S1, S2, S3, S5], Leptotrichia [S1, S3], Listeria [S10], Mageeibacillus [S1, S5], Mycobacterium [S7], Mycoplasma [S7], Neisseria [S12], Oenococcus [S12] , Oceanivirga [S1], Paeniglutamicibacter [S3], Parvimonas [S1], Pasteurella [S13], Petrimonas [S5], Porphyromonas [S1], Sneathia [S5], Staphylococcus [S3], Streptococcus [S3], Toxoplasma [S8]
Species	Pregnant	Bifidobacterium breve [S14], Enterococcus faecium [S15], Peptostreptococcus anaerobius [S16, S17]
	Non-pregnant	Actinobacillus seminis [S5], Actinomyces israelii [S18], Bacteroides fragilis [S19], Brucella abortus [S20], Brucella melitensis [S7], Brucella ovis [S21], Brucella suis [S7], Campylobacter fetus [S7, S9], Campylobacter jejuni [S9], Candida albicans [S22], Candida glabrata [S23], Chlamydia abortus [S7], Chlamydia psittaci [S7, S8], Chlamydia trachomatis [S7], Clostridium perfringens [S24], Coxiella burnetii [S7], Erysipelothrix rhusiopathiae [S25], Escherichia coli [S26], Fusobacterium necrophorum [S27, S28], Fusobacterium nucleatum [S29], Gardnerella vaginalis [S30], Haemophilus somnus [S31], Helcococcus ovis [S32], Histophilus somni [S1], Leptospira hardjo [S11, S33], Leptospira interrogans [S33], Leptotrichia amnionii [S34], Leptotrichia sanguinegens [S5], Listeria monocytogenes [S35], Mageeibacillus indolicus [S5], Mycoplasma agalactiae [S7], Mycoplasma hominis [S36], Neospora caninum [S7], Pasteurella haemolytica [S37], Pasteurella multocida [S13], Porphyromonas levii [S38], Prevotella bivia [S16, S39], Prevotella nigrescens [S40], Sneathia sanguinegens [S5], Staphylococcus aureus [S41], Streptococcus agalactiae [S42], Toxoplasma gondii [S7, S8], Trichomonas foetus [S43], Trichomonas vaginalis [S44], Trueperella pyogenes [S45, S46], Ureaplasma diversum [S5], Ureaplasma urealyticum [S47]

High Abundance: indicates the group in which the taxon was reported as more abundant in the cited literature. **Level**: denotes the taxonomic rank at which the association was reported. The table compiles prior evidence only and does not present results from the current study.

Reference

S1. Reinoso-Peláez EL, Puente-Sánchez F, Serrano M, Calvo JH, Ramon M, Saura M. Characterization of bacterial communities of ewe's vaginal tract and its potential impact on reproductive efficiency. Anim Microbiome. 2025;7:1–14. https://doi.org/10.1186/s42523-025-00383-2.

S2. Reinoso-Peláez EL, Saura M, González C, Ramón M, Calvo JH, Serrano M. The influence of vaginal microbiota on ewe fertility: a metagenomic and functional genomic approach. Microbiome. 2025. https://doi.org/10.1186/s40168-025-02165-z.

S3. Koester LR, Petry AL, Youngs CR, Schmitz-Esser S. Ewe vaginal microbiota: associations with pregnancy outcome and changes during gestation. Front Microbiol. 2021;12:745884. https://doi.org/10.3389/FMICB.2021.745884.

S4. Barba M, Toquet M, García-Roselló E, Gomis J, Quereda JJ, González-Torres P, et al. Description of the vaginal microbiota in nulliparous ewes during natural mating and pregnancy: preliminary signs of the male preputial microbiota modulation. Front Microbiol. 2024;14:1224910. https://doi.org/10.3389/FMICB.2023.1224910.

S5. Serrano M, Climent E, Freire F, Martínez-Blanch JF, González C, Reyes L, et al. Influence of the ovine genital tract microbiota on the species artificial insemination outcome. A pilot study in commercial sheep farms. High Throughput. 2020;9:16. https://doi.org/10.3390/ht9030016.

S6. Quereda JJ, García-Roselló E, Barba M, Mocé ML, Gomis J, Jiménez-Trigos E, et al. Use of probiotics in intravaginal sponges in sheep: A pilot study. Animals. 2020;10:719. https://doi.org/10.3390/ANI10040719.

S7. Ruiz-Fons F, González-Barrio D, Aguilar-Ríos F, Soler AJ, Garde JJ, Gortázar C, et al. Infectious pathogens potentially transmitted by semen of the black variety of the Manchega sheep breed: health constraints for conservation purposes. Anim Reprod Sci. 2014;149:152–7. https://doi.org/10.1016/J.ANIREPROSCI.2014.07.006.

S8. Kirkbride CA. Diagnoses in 1,784 ovine abortions and stillbirths. J Vet Diagn Invest. 1993;5:398–402. https://doi.org/10.1177/104063879300500316.

S9. Yaeger MJ, Sahin O, Plummer PJ, Wu Z, Stasko JA, Zhang Q. The pathology of natural and experimentally induced Campylobacter jejuni abortion in sheep. Journal of Veterinary Diagnostic Investigation. 2021;33:1096–105. https://doi.org/10.1177/10406387211033293.

S10. Poole RK, Soffa DR, McAnally BE, Smith MS, Hickman-Brown KJ, Stockland EL. Reproductive microbiomes in domestic livestock: insights utilizing 16S rRNA gene amplicon community sequencing. Animals. 2023;13:485. https://doi.org/10.3390/ani13030485.

S11. Smith KE, Garza AL, Robinson C, Ashley RL, Ivey SL. 1039 WS Influence of sampling location and pregnancy on composition of the microbiome associated with the reproductive tract of the ewe. Journal of Animal Science. 2016;94 suppl_5:498–498. https://doi.org/10.2527/jam2016-1039.

S12. Reinoso-Peláez EL, Saura M, González-Recio Ó, González C, Fernández A, Peiro-Pastor R, et al. Impact of oestrus synchronization devices on ewes vaginal microbiota and artificial insemination outcome. Front Microbiol. 2023;14:1063807. https://doi.org/10.3389/FMICB.2023.1063807.

S13. Watson PJ, Davies RL. Outbreak of Pasteurella multocida septicaemia in neonatal lambs. Veterinary Record. 2002;151:420–2. https://doi.org/10.1136/vr.151.14.420.

S14. Bottacini F, O'Connell Motherway M, Kuczynski J, O'Connell KJ, Serafini F, Duranti S, et al. Comparative genomics of the Bifidobacterium breve taxon. BMC Genomics. 2014;15:1–19. https://doi.org/10.1186/1471-2164-15-170.

S15. Shallal EN, Asker AS, Yaseen AA. Isolation and Identification of Enterococcus faecium Bacteria From the Vaginal Cavity of Local Ewes and Study of some of their Antagonistic Properties Against Types of Pathogenic Bacteria. IOP Conf Ser Earth Environ Sci. 2024;1371:072004. https://doi.org/10.1088/1755-1315/1371/7/072004.

S16. Pybus V, Onderdonk AB. A commensal symbiosis between Prevotella bivia and Peptostreptococcus anaerobius involves amino acids: potential significance to the pathogenesis of bacterial vaginosis. FEMS Immunol Med Microbiol. 1998;22:317–27. https://doi.org/10.1111/J.1574-695X.1998.TB01221.X.

S17. Legaria MC, Nastro M, Camporro J, Heger F, Barberis C, Stecher D, et al. Peptostreptococcus anaerobius: Pathogenicity, identification, and antimicrobial susceptibility. Review of monobacterial infections and addition of a case of urinary tract infection directly identified from a urine sample by MALDI-TOF MS. Anaerobe. 2021;72:102461. https://doi.org/10.1016/J.ANAEROBE.2021.102461.

S18. Evans DTP. Actinomyces israelii in the female genital tract: a review. Genitourin Med. 1993;69:54. https://doi.org/10.1136/STI.69.1.54.

S19. Evaldson G, Malmborg AS, Nord CE, Östensson K. Bacteroides fragilis, Streptococcus intermedius and group B streptococci in ascending infection of pregnancy: an animal experimental study. Gynecol Obstet Invest. 1983;15:230–41. https://doi.org/10.1159/000299415.

S20. Ocholi R, Kwaga J, Ocholi RA, Kwaga JKP, Ajogi I, Bale JOO. Abortion due to Brucella abortus in sheep in Nigeria. 2005.

S21. Braz HMB, Silva MF, Carvalho TP de, Silva LA da, Soares JB, Costa FB, et al. Pathogenesis of Brucella ovis in pregnant mice and protection induced by the candidate vaccine strain B. Ovis ΔabcBA. Vaccine. 2022;40:4617–24. https://doi.org/10.1016/J.VACCINE.2022.06.044.

S22. Stock SJ, Patey O, Thilaganathan B, White S, Furfaro LL, Payne MS, et al. Intrauterine Candida albicans Infection Causes Systemic Fetal Candidiasis With Progressive Cardiac Dysfunction in a Sheep Model of Early Pregnancy. https://doi.org/101177/1933719116649697. 2016;24:77–84. https://doi.org/10.1177/1933719116649697.

S23. Asemota OA, Nyirjesy P, Fox R, Sobel JD. Candida glabrata complicating in vitro pregnancy: successful management of subsequent pregnancy. Fertil Steril. 2011;95:803.e1-803.e2. https://doi.org/10.1016/J.FERTNSTERT.2010.07.1095.

S24. Fthenakis GC, Arsenos G, Brozos C, Fragkou IA, Giadinis ND, Giannenas I, et al. Health management of ewes during pregnancy. Anim Reprod Sci. 2012;130:198–212. https://doi.org/10.1016/J.ANIREPROSCI.2012.01.016.

S25. Ersdal C, Jørgensen HJ, Lie KI. Acute and Chronic Erysipelothrix rhusiopathiae Infection in Lambs. Vet Pathol. 2015;52:635–43. https://doi.org/10.1177/0300985814556187/ASSET/IMAGES/LARGE/10.1177_0300985814556187-FIG3.JPEG.

S26. Sargison ND, Howie F, Mearns R, Penny CD, Foster G. Shiga toxin-producing Escherichia coli as a perennial cause of abortion in a closed flock of Suffolk ewes. Veterinary Record. 2007;160:875–6. https://doi.org/10.1136/vr.160.25.875.

S27. Bicalho MLS, Machado VS, Oikonomou G, Gilbert RO, Bicalho RC. Association between virulence factors of Escherichia coli, Fusobacterium necrophorum, and Arcanobacterium pyogenes and uterine diseases of dairy cows. Vet Microbiol. 2012;157:125–31. https://doi.org/10.1016/J.VETMIC.2011.11.034.

S28. Souza NC, Ramos TNM, Borsanelli AC, Saraiva JR, Ferreira EM, Schweitzer CM, et al. Monitoring periodontal lesions and their effects during pregnancy: microbiological aspects of the oral cavity and amniotic fluid in pregnant ewes. Pesquisa Veterinária Brasileira. 2023;43:e07160. https://doi.org/10.1590/1678-5150-PVB-7160.

S29. Vander Haar EL, So J, Gyamfi-Bannerman C, Han YW. Fusobacterium nucleatum and adverse pregnancy outcomes: epidemiological and mechanistic evidence. Anaerobe. 2018;50:55–9. https://doi.org/10.1016/J.ANAEROBE.2018.01.008.

S30. Catlin BW. Gardnerella vaginalis: characteristics, clinical considerations, and controversies. Clin Microbiol Rev. 1992;5:213–37. https://doi.org/10.1128/CMR.5.3.213.

S31. Kwiecien JM, Little PB. Haemophilus somnus and reproductive disease in the cow: A review. The Canadian Veterinary Journal. 1991;32:595.

S32. Locatelli C, Scaccabarozzi L, Pisoni G, Bronzo V, Casula A, Testa F, et al. Helcococcus kunzii and Helcococcus ovis isolated in dairy cows with puerperal metritis. J Gen Appl Microbiol. 2013;59:371–4. https://doi.org/10.2323/JGAM.59.371.

S33. Director A, Penna B, Hamond C, Loureiro AP, Martins G, Medeiros MA, et al. Isolation of Leptospira interrogans Hardjoprajitno from vaginal fluid of a clinically healthy ewe suggests potential for venereal transmission. J Med Microbiol. 2014;63 PART 9:1234–6. https://doi.org/10.1099/JMM.0.065466-0/CITE/REFWORKS.

S34. Gruwier L, Sprenkels A, Hulsbosch S, Vankeerberghen A, Cartuyvels R. Sneathia amnii bacteraemia and chorioamnionitis leading to second trimester abortion: a case report. Access Microbiol. 2021;3:000290. https://doi.org/10.1099/ACMI.0.000290.

S35. Brugère-Picoux J. Ovine listeriosis. Small Ruminant Research. 2008;76:12–20. https://doi.org/10.1016/J.SMALLRUMRES.2007.12.022.

S36. Hosny AEDMS, El-khayat W, Kashef MT, Fakhry MN. Association between preterm labor and genitourinary tract infections caused by Trichomonas vaginalis, Mycoplasma hominis, Gram-negative bacilli, and coryneforms. Journal of the Chinese Medical Association. 2017;80:575–81. https://doi.org/10.1016/J.JCMA.2016.10.007.

S37. Ward AC. Isolation of Pasteurellaceae from Bovine Abortions. https://doi.org/101177/104063879000200111. 1990;2:59–62. https://doi.org/10.1177/104063879000200111.

S38. Elad D, Friedgut O, Alpert N, Stram Y, Lahav D, Tiomkin D, et al. Bovine necrotic vulvovaginitis associated with Porphyromonas levii. Emerg Infect Dis. 2004;10:505. https://doi.org/10.3201/EID1003.020592.

S39. Gilbert NM, Lewis WG, Li G, Sojka DK, Lubin JB, Lewis AL. Gardnerella vaginalis and Prevotella bivia Trigger Distinct and Overlapping Phenotypes in a Mouse Model of Bacterial Vaginosis. J Infect Dis. 2019;220:1099–108. https://doi.org/10.1093/INFDIS/JIY704.

S40. Huang Z, DerGarabedian BP, He L, Sha Y, Chen Z, Kang J, et al. Impact of periodonto-pathogenic microbiota and sociodemographic variables on periodontal status during pregnancy and postpartum period. Oral Health Prev Dent. 2020;18:855–64. https://doi.org/10.3290/j.ohpd.a45355.

S41. Martinez-Ros P, Lozano M, Hernandez F, Tirado A, Rios-Abellan A, López-Mendoza MC, et al. Intravaginal device-type and treatment-length for ovine estrus synchronization modify vaginal mucus and microbiota and affect fertility. Animals. 2018;8:226. https://doi.org/10.3390/ANI8120226.

S42. Santana FAF, de Oliveira TVL, Filho MB de S, da Silva LSC, de Brito BB, de Melo FF, et al. Streptococcus agalactiae: Identification methods, antimicrobial susceptibility, and resistance genes in pregnant women. World J Clin Cases. 2020;8:3988. https://doi.org/10.12998/WJCC.V8.I18.3988.

S43. Agnew DW, Munson L, Cobo ER, Olesen D, Corbeil LB, BonDurant RH. Comparative histopathology and antibody responses of non-Tritrichomonas foetus trichomonad and Tritrichomonas foetus genital infections in virgin heifers. Vet Parasitol. 2008;151:170–80. https://doi.org/10.1016/J.VETPAR.2007.10.016.

S44. Edwards T, Burke P, Smalley H, Hobbs G. Trichomonas vaginalis: Clinical relevance, pathogenicity and diagnosis. Crit Rev Microbiol. 2016;42:406–17. https://doi.org/10.3109/1040841X.2014.958050.

S45. Bicalho MLS, Lima FS, Machado VS, Meira EB, Ganda EK, Foditsch C, et al. Associations among Trueperella pyogenes, endometritis diagnosis, and pregnancy outcomes in dairy cows. Theriogenology. 2016;85:267–74. https://doi.org/10.1016/J.THERIOGENOLOGY.2015.09.043.

S46. Rzewuska M, Kwiecień E, Chrobak-Chmiel D, Kizerwetter-Świda M, Stefańska I, Gieryńska M. Pathogenicity and virulence of Trueperella pyogenes: a review. Int J Mol Sci. 2019;20:2737. https://doi.org/10.3390/IJMS20112737.

S47. Cassell GH, Waites KB, Watson HL, Crouse DT, Harasawa R. Ureaplasma urealyticum intrauterine infection: role in prematurity and disease in newborns. Clin Microbiol Rev. 1993;6:69–87. https://doi.org/10.1128/CMR.6.1.69.
